# Neonatal brain MRI and short-term outcomes after acute provoked seizures

**DOI:** 10.1038/s41372-023-01723-3

**Published:** 2023-07-15

**Authors:** Yi Li, Aaron Scheffler, Anthony James Barkovich, Taeun Chang, Catherine J. Chu, Shavonne L. Massey, Nicholas S. Abend, Monica E. Lemmon, Cameron Thomas, Adam Numis, Linda S. Franck, Elizabeth Rogers, Andrew Callen, Charles E. McCulloch, Renée A. Shellhaas, Hannah C. Glass

**Affiliations:** 1 Department of Radiology and Biomedical Imaging, University of California San Francisco, San Francisco, CA USA; 2 Department of Epidemiology and Biostatistics, University of California San Francisco, San Francisco, CA USA; 3 Department of Neurology, Children’s National Hospital, George Washington University School of Medicine, Washington, DC USA; 4 Department of Neurology, Massachusetts General Hospital, Harvard Medical School, Boston, MA USA; 5 Departments of Neurology and Pediatrics, Children’s Hospital of Philadelphia and Perelman School of Medicine at the University of Pennsylvania, Philadelphia, PA USA; 6 Departments of Anesthesia & Critical Care Medicine, Children’s Hospital of Philadelphia and Perelman School of Medicine at the University of Pennsylvania, Philadelphia, PA USA; 7 Department of Pediatrics and Population Health Sciences, Duke University School of Medicine, Durham, NC USA; 8 Department of Pediatrics, University of Cincinnati and Division of Neurology, Cincinnati Children’s Hospital Medical Center, Cincinnati, OH USA; 9 Department of Neurology and Weill Institute for Neuroscience, University of California San Francisco, San Francisco, CA USA; 10 Department of Family Health Care Nursing, UCSF Benioff Children’s Hospital, University of California San Francisco, San Francisco, CA USA; 11 Department of Pediatrics, UCSF Benioff Children’s Hospital, University of California San Francisco, San Francisco, CA USA; 12 Department of Radiology, University of Colorado Denver School of Medicine, Denver, CO USA; 13 Division of Pediatric Neurology, Department of Neurology, Washington University in St. Louis, St. Louis, MO USA

**Keywords:** Outcomes research, Paediatric neurological disorders

## Abstract

**Objective:**

We investigated how diagnosis and injury location on neonatal brain MRI following onset of acute provoked seizures was associated with short term outcome.

**Study design:**

A multicenter cohort of neonates with acute provoked seizures enrolled in the *Neonatal Seizure Registry*. MRIs were centrally evaluated by a neuroradiologist for location of injury and radiologic diagnosis. Clinical outcomes were determined by chart review. Multivariate logistic regression was used to examine the association between MRI findings and outcomes.

**Results:**

Among 236 newborns with MRI at median age 4 days (IQR 3–8), 91% had abnormal MRI. Radiologic diagnoses of intracranial hemorrhage (OR 3.2 [1.6–6.5], *p* < 0.001) and hypoxic-ischemic encephalopathy (OR 2.7 [1.4–5.4], *p* < 0.003) were associated with high seizure burden. Radiologic signs of intracranial infection were associated with abnormal neurologic examination at discharge (OR 3.9 [1.3–11.6], *p* < 0.01).

**Conclusion:**

Findings on initial MRI can help with expectant counseling on short-term outcomes following acute provoked neonatal seizures.

## Introduction

Neonatal acute symptomatic or acute provoked seizures, defined as seizures due to acute brain injury, affect approximately 1–3 out of every 1000 live births in the United States each year [1, 2]. Acute provoked seizures are often the first sign of neurologic dysfunction in neonates who have suffered acute brain injury. The most common causes of seizures in neonates are acute brain injury due to hypoxic-ischemic encephalopathy (HIE), ischemic stroke, or intracranial hemorrhage [3]. Children with acute provoked seizures are at high risk of adverse neurodevelopmental outcome, including post-neonatal epilepsy, developmental delay, intellectual disability, and cerebral palsy [1]. Despite advances in neonatal neurocritical care, relatively little is known about the risk factors that predispose some neonates to experience a higher seizure burden or abnormal neurologic examination at hospital discharge after acute provoked seizures.

Magnetic resonance imaging (MRI) is increasingly used to determine seizure etiology and predict long-term prognosis for neonates with seizures, but little is known about the relationship between seizures and specific neuroimaging findings in this population. Prior studies have been limited by single center enrollment [1, 4–8]. A detailed understanding of the relationship between early imaging findings and short-term clinical outcomes has the potential to guide parent counseling during the neonatal period.

We evaluated a large, prospective multicenter cohort of infants with acute provoked seizures enrolled in the *Neonatal Seizure Registry* to test the hypothesis that the etiology and location of brain injury detected on early neonatal brain MRI following onset of acute provoked seizures are associated with seizure burden and neurologic outcomes at hospital discharge.

## Methods

### Study design

This was an ancillary study of a prospective, multicenter cohort of infants with acute provoked neonatal seizures born between 7/2015 and 3/2018 who survived the neonatal admission, enrolled at seven *National Seizure Registry (NSR)* sites (NCT02789176) [9, 10]. Each site has a level IV neonatal intensive care unit (NICU) and followed the American Clinical Neurophysiology Society (ACNS) guidelines for continuous electroencephalographic monitoring (cEEG) in neonates [11]. The local institutional review board at each site approved the study, and neonates were enrolled after informed parental consent.

### Inclusion and exclusion criteria

**Fig. 1:**
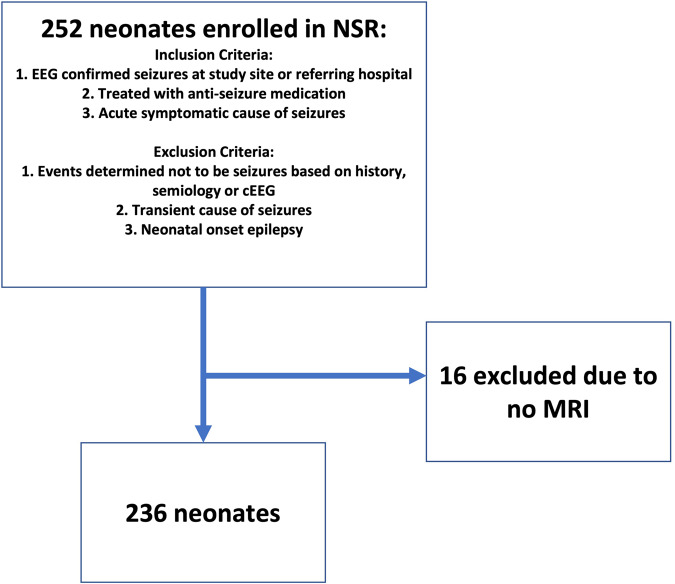
Inclusion and exclusion criteria for the study. After exclusion of 16 neonates with no MRI, the final cohort consisted of 236 neonates.

*NSR* enrollment was determined at each site by the study site investigator, a pediatric neurologist. Enrollment criteria were pre-established (Fig. 1): (1) neonate with EEG-confirmed seizure at either the study site or the referring hospital, or (2) neonate treated with antiseizure medication for suspected clinical seizures with a clinical history, including event semiology, supporting the diagnosis of seizures, and (3) seizures were due to an acute symptomatic cause. Neonates were excluded if events were determined *not* to be seizures based on history, semiology, or cEEG, or were determined not to be transient or secondary to an acute provoked cause (i.e., genetic or neonatal epilepsy syndromes). Neonates were also excluded from the present ancillary analysis if there was no available brain MRIs or if the brain MRI was of nondiagnostic quality. Only children who survived the neonatal admission were enrolled.

### Measurements

#### Clinical and demographic data

Clinical information was obtained prospectively upon enrollment by chart review performed at enrollment sites, performed by the study team led by the site investigator, a pediatric neurologist. Clinical information included sex, preterm birth (defined as less than 37 weeks gestation at birth), and complex medical course (defined binarily by any combination of congenital heart disease, cardiac failure, extracorporeal membrane oxygenation treatment, or dialysis).

#### MRI

All MRI studies were performed using local institutional protocols. The first MRI after neonatal seizure onset was centrally reviewed by a board-certified, fellowship-trained pediatric neuroradiologist with 7 years of experience (YL). The first ten MRIs were also reviewed by a pediatric neuroradiologist with 40 years of experience (AJB) to establish consensus in scoring methodology.

Studies that were not of diagnostic quality, based on the degree of motion degradation, were identified and excluded. Studies were evaluated for available sequences [T1, T2, Diffusion Weighted Imaging (DWI), Susceptibility Weighted Imaging (SWI), and T1 post-contrast].

**Fig. 2:**
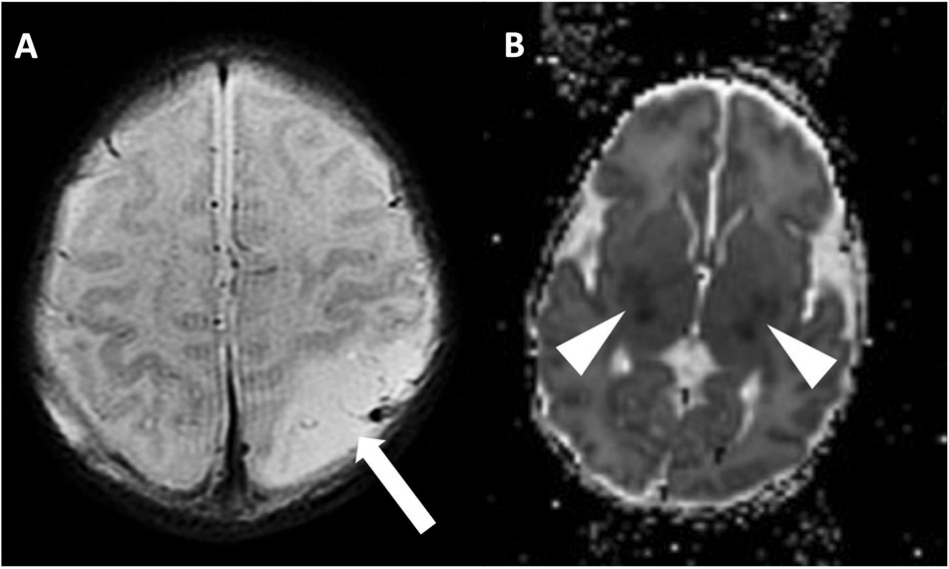
Examples of injury based on location. **A** Axial T2 weighted imaging demonstrating cortical injury in the setting of arterial ischemic stroke, with missing cortical ribbon (white arrow) in area of edematous, injured cortex. **B** Axial ADC map demonstrating reduced diffusion in the deep gray nuclei signifying acute injury (arrowheads).

In generating the primary imaging predictors, a binary scoring system (present/absent) was used to assess for any signal abnormality corresponding to injury involving cortex and deep gray nuclei (Fig. 2). Examples of cortical injury include reduced diffusion signifying acute ischemia, T1 hyperintensity compatible with laminar necrosis, and loss of the cortical ribbon compatible with cortical edema. Examples of deep gray injury include reduced diffusion signifying acute ischemia, susceptibility artifact compatible with hemorrhage, abnormal T1 hyperintensity or T2 hypointensity signifying evolving injury in the setting of HIE, and T2 hyperintensity signifying edema.

**Fig. 3:**
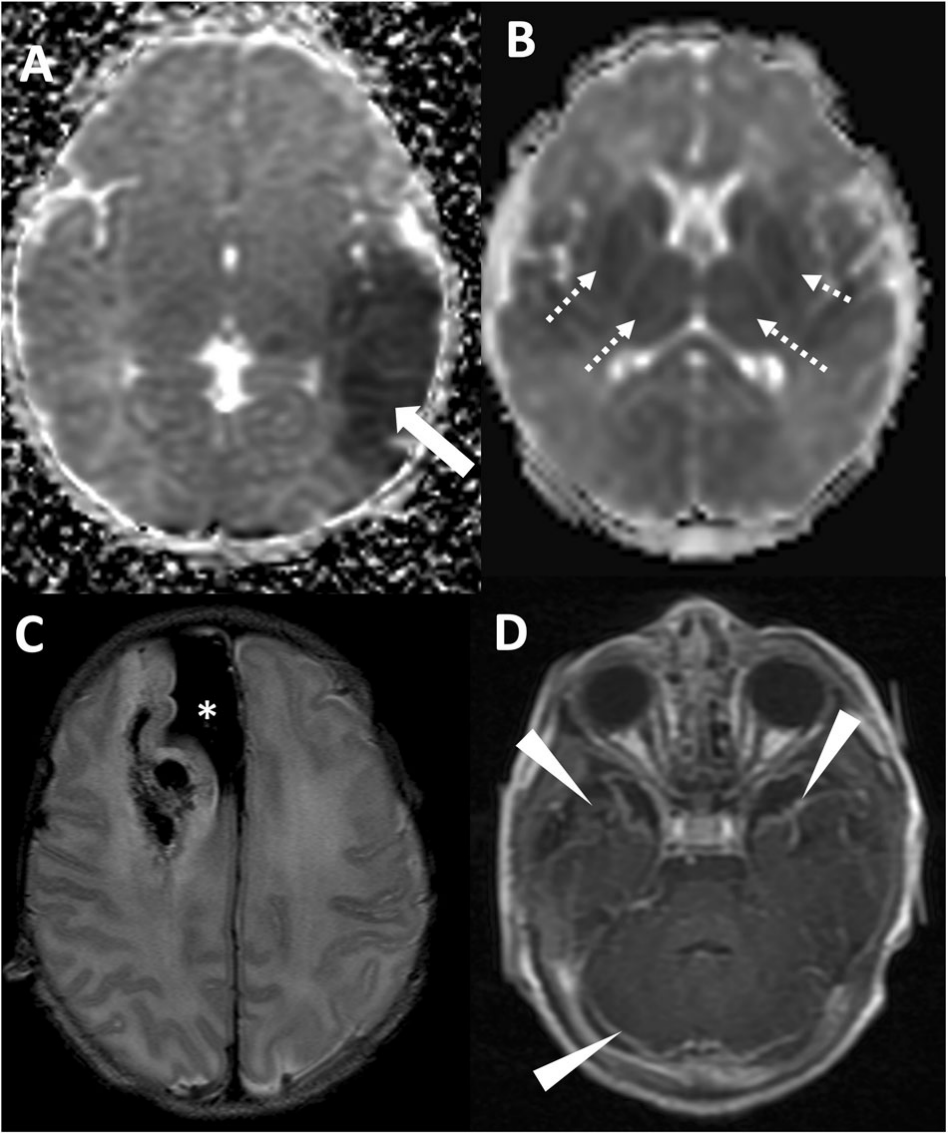
Radiologic diagnoses in the cohort of patients with acute symptomatic seizures. **A** Axial ADC map demonstrating arterial ischemic stroke in the left middle cerebral artery territory (white arrow). **B** Axial ADC map demonstrating reduced diffusion in the deep gray nuclei and subcortical white matter, compatible with hypoxic ischemic injury. **C** Axial T2 weighted imaging demonstrating large subpial hemorrhage in the medial right frontal lobe (asterisk) with associated hemorrhagic injury of the right frontal lobe. **D** Axial T1-weighted post-contrast imaging demonstrating leptomeningeal enhancement (arrowheads) in the setting of neonatal meningitis/intracranial infection.

A similar binary model (present/absent) was used to categorize MRI studies regarding specific radiologic diagnoses including: normal, HIE, focal ischemic stroke (arterial, venous, or other etiology), intracranial hemorrhage (ICH), suspected infection (based on findings of leptomeningeal enhancement, and reduced diffusion signifying abscess, empyema or ventriculitis), and “other” (including suspected hypoglycemia, chronic injury including encephalomalacia, low white matter volume, and periventricular white matter injury of prematurity) (Fig. 3). Small parturitional subdural hematoma was not scored as abnormality, as it was felt to be common and not contributory to seizure etiology. Neonates could have more than one radiologic diagnosis and could have injury in none, one, or both scored locations.

#### Clinical outcomes data

The primary clinical seizure etiology was determined by the enrolling site investigator based on a combination of clinical and radiologic factors and categorized as follows: HIE, ischemic stroke, ICH, infection, hypoglycemia, and other.

Two primary clinical outcomes of interest were collected by the site investigator for the purposes of this analysis. First, EEG-seizure burden was assessed on a 5-point scale[3]: None, rare (<7), many isolated (> = 7), frequent recurrent EEG seizures, status epilepticus [12], or documentation inadequate to quantify. For analysis, these categories were dichotomized into low seizure burden (defined as rare seizures), and high seizure burden (defined as many isolated, frequent recurrent EEG seizures, or status epilepticus) [10]. Second, neurologic examination at time of discharge was determined by the site investigator as normal or abnormal (defined as any abnormality in consciousness, tone, or reflexes).

Two additional secondary outcomes of interest were evaluated: (1) presence of EEG-only seizures, defined as EEG seizures without recognized clinical correlate, and (2) incomplete response to loading dose of antiseizure medication, defined as ongoing seizures after 30 min despite a loading dose of antiseizure medication (defined as at least 20 mg/kg for phenobarbital and phenytoin/fosphenytoin and 40 mg/kg for levetiracetam).

### Statistical analysis

In multiple logistic regression, imaging predictors including location of injury and radiologic diagnosis were modeled in association with the outcomes of seizure burden and abnormal discharge examination, while controlling for clinically identified potential confounders of sex, preterm birth, and complex medical course. In multivariate analysis, predictors reaching statistical significance of *p* < 0.1 were modeled in association with outcomes using logistic regression, also controlling for clinically identified potential confounders of sex, preterm birth, and complex medical course.  We used backward elimination of variables with *p* < 0.05 to arrive at our final, multivariate reported model.

Analyses and adjustments were conducted using Stata version 16 (College Station, TX) [13].

## Results

### Demographics and MRI diagnoses

Two hundred fifty-two newborns were enrolled. Sixteen newborns were excluded from the current analysis due to incomplete imaging data. Table 1 provides the demographic information for the 236 infants included in this analysis.

**Table 1:** Clinical characteristics for 236 prospectively enrolled neonates with acute provoked seizures and clinical brain MRI.

	*N* = 236
Male sex	130 (55%)
Gestational age, weeks	39.3 (38.1–40.4)
Birth weight, kg	3.1 (±0.7 kg)
Apgar at 1, 5 min	4 (2–8), 8 (5–9)
Therapeutic hypothermia	70 (30%)
Therapeutic hypothermia in neonates with radiologic HIE	26/67 (39%)
Complex medical course (cardiac, congenital heart disease, ECMO, dialysis)	25 (11%)
Seizure burden at the study center	
None	40 (17%)
Rare EEG seizures (<7)	65 (28%)
Many isolated EEG seizures (> = 7)	49 (21%)
Frequent recurrent EEG seizures	44 (19%)
Status epilepticus	37 (16%)
Unknown	1 (0.4%)
EEG-only seizures	44 (19%)
Incomplete response to loading dose of anti-seizure medication	149 (63%)
Abnormal neurologic examination at discharge	73 (31%)

Data are presented as *n* (%) and mean (±standard deviation) or median (interquartile range).

MRI was performed at a median age 4 (IQR 3 to 8) days after birth. No studies were nondiagnostic in quality. MRIs contained the following sequences: T2 99%, DWI 97%, T1 94%, SWI 89%, post-contrast T1 26%.

Cortical injury was present in 118/236 (50%) neonates, and deep gray injury was present in 89/236 (38%).

Radiologic etiologies included focal ischemic stroke in 85 (36%), HIE in 67 (28%), ICH in 22 (9%), suspected infection in 16 (7%), and other etiologies in 30 (12%). Twenty-two (9%) had a normal MRI. More than one radiologic diagnosis was present in 42 (18%). Fourteen of 22 (64%) neonates with a normal MRI had a clinical diagnosis of HIE as the underlying seizure etiology. Among these 14 neonates with normal MRI and HIE, 13 (93%) had undergone therapeutic hypothermia. Supplementary Table 1 provides the distribution of radiologic diagnoses, in comparison to primary clinical diagnoses.

### MRI findings and primary outcomes

#### Seizure burden

Neonates with a normal MRI were less likely to have a high seizure burden (3/22, 14%) compared to those with an abnormal MRI (127/214, 59%, OR 0.13 [0.04–0.45], *p* < 0.001, Table 2). A high seizure burden was more likely among children with a radiologic diagnosis of HIE (67% with HIE vs 50% without HIE, OR 2.4 [1.3–4.4], *p* < 0.006) and less likely among those with a radiologic diagnosis of “other” (39% with “other” diagnoses vs 59% with HIE, ischemic stroke, intracranial hemorrhage, or intracranial infection, OR 0.44 [0.22–0.85], *p* < 0.02). Neonates with ICH had a trend toward high seizure burden (68% with ICH vs 51% without ICH, OR 1.8 [0.96–3.4], *p* < 0.07). Cortical injury was significantly associated with a high seizure burden (68% with cortical injury vs 42% without cortical injury, OR 3.5 [1.9–6.4], *p* < 0.001). Deep gray injury was also associated with a high seizure burden (63% with deep gray injury vs 50% without deep gray injury, OR 1.7 [1.0–3.0], *p* = 0.045).

In the multivariate model, a diagnosis of HIE (OR 2.7 [1.4–5.4], *p* < 0.003), ICH (OR 3.2 [1.6–6.5], *p* < 0.001), or cortical injury (OR 3.5 [1.9–6.4], *p* < 0.001) remained independently associated with high seizure burden.

#### Neurologic examination at discharge

Neonates with MRI evidence of HIE were more likely to have an abnormal neurologic examination at discharge (42% with HIE vs 27% without HIE, OR 2.2 [1.1–3.7], *p* < 0.02). Neonates with a radiologic diagnosis of infection were also more likely to have an abnormal neurologic examination at discharge (63% with infection vs 29% without infection, OR 4.2 [1.4–12.0], *p* < 0.008). Deep gray injury on early MRI was also associated with abnormal discharge examination (45% with deep gray injury vs 22% without deep gray injury, OR 2.8 [1.6–5.0], *p* < 0.001).

In the multivariate model, a radiologic diagnosis of infection (OR 3.9 [1.3–11.6], *p* < 0.01) and deep gray injury (OR 2.7 (1.6–5.0], *p* < 0.001) remained independently associated with abnormal neurologic examination at discharge.

**Table 2:** Odds of high seizure burden and abnormal neurological examination at discharge based on radiologic diagnosis and injury location in 236 children with acute provoked neonatal seizures.

MRI Findings	High Seizure Burden		Abnormal Neuro Examination at Discharge	
	Minimally Adjusted*	Fully Adjusted/Multivariate*	Minimally Adjusted*	Fully Adjusted/Multivariate*
Imaging Diagnoses				
Normal MRI (*N* = 22)	**0.13 [0.04–0.45],** ***p*** < **0.001**		0.49 [0.16–1.5], *p* < 0.21	
HIE (*N* = 67)	**2.4 [1.3–4.4],** ***p*** < **0.006**	**2.7 [1.4–5.4],** ***p*** < **0.003**	**2.2 [1.1–3.7],** ***p*** < **0.02**	
Ischemic Stroke (*N* = 84)	0.90 [0.52–1.6], *p* < 0.71		0.7 [0.4–1.3], *p* < 0.30	
Intracranial Hemorrhage (*N* = 62)	1.8 [0.96–3.4], *p* < *0.07*	**3.2 [1.6–6.5],** ***p*** < **0.001**	0.9 [0.45–1.7], *p* < 0.67	
Infection (*N* = 6)	2.5 [0.76–8.1], *p* < 0.13		**4.2 [1.4–12.0],** ***p*** < **0.008**	**3.9 [1.3–11.6],** ***p*** < **0.01**
Other diagnosis (*N* = 51)	**0.44 [0.22–0.85],** ***p*** < **0.02**		0.75 [0.4–1.5], *p* < 0.43	
Injury Location				
Cortical Injury (*N* = 118)	**2.9 [1.7–5.1],** ***p*** < **0.001**	**3.5 [1.9-6.4],** ***p*** < **0.001**	1.5 [0.86–2.7], *p* < 0.16	
Deep Gray Injury (*N* = 89)	**1.7 [1.0–3.0],** ***p*** < **0.05**		**2.8 [1.6–5.0],** ***p*** < **0.001**	**2.7 [1.6–5.0],** ***p*** < **0.001**
Other Injury Location (*N* = 61)	0.64 [0.35–1.2], *p* < 0.15		0.51 [0.26–1.03], *p* < 0.06	

Data are presented as odd ratio [95% confidence interval)

*Adjusted for sex, preterm birth, and complex medical course

Bold values indicate statistical significance *p* < 0.05

### MRI findings and secondary outcomes

#### EEG-only seizures

Neonates with ischemic stroke were less likely to have EEG-only seizures (11% with stroke vs 23% without stroke, OR 0.42 [0.5–0.9], *p* < 0.03, Supplementary Table 2). Cortical injury was also negatively associated with EEG-only seizures (7% with cortical injury vs 30% without cortical injury, OR 0.2 [0.09–0.45], *p* < 0.0001).

In the multivariate model, the negative association between cortical injury and EEG-only seizures remained statistically significant (OR 0.2 [0.09–0.45], *p* < 0.0001).

#### Treatment resistant seizures

Neonates with a normal MRI were less likely to have treatment resistant seizures (22% with normal MRI compared to 67% with abnormal MRI, OR 0.13 [0.05–0.38], *p* < 0.0001). Among the radiologic diagnoses, HIE was significantly associated with treatment resistant seizures (84% with HIE vs 55% without HIE, OR 5.2 [2.4–11.5], *p* < 0.0001). Cortical injury was significantly associated with treatment resistant seizures (73% with cortical injury vs 53% without cortical injury, OR 2.6 [1.5–4.7], *p* < 0.001).

In the multivariate model, a diagnosis of HIE (OR 4.9 [2–10.8], *p* < 0.0001) and cortical injury (OR 4.9 [2.2–10.8], *p* < 0.001) remained associated with treatment resistant seizures.

## Discussion

In this large, prospective, multi-center cohort of neonates with acute provoked seizures and MRI at a median of 4 days, specific imaging abnormalities were associated with short-term clinical outcomes and seizure characteristics. These findings can be used to guide parental counseling in the neonatal period. Ninety one percent of neonates with acute provoked seizures had an abnormal brain MRI. In the multivariate analysis, HIE and ICH were associated with high seizure burden, and HIE was associated with treatment resistant seizures. MRI evidence of intracranial infection was associated with an abnormal neurologic examination at discharge. With regards to location of injury, cortical injury was associated with higher seizure burden and treatment resistant seizures, while deep gray injury was associated with higher odds of an abnormal neurologic examination at discharge. These imaging-focused data add further detail to the existing knowledge about neonates with acute provoked seizures [3], allowing for more detailed counseling on short-term outcomes based on specific imaging diagnoses and locations of injury.

In multivariate analysis, ICH was associated with highest odds of high seizure burden. Prior studies have found that acute symptomatic seizures may be associated with any form of intracranial hemorrhage, whether intra- or extra-axial, although parturitional subdural hemorrhage, which was not scored in this study, is not typically associated with seizures [14]. In one study, seizure rates did not differ based on traumatic versus non-traumatic cause of intracranial hemorrhage, but parenchymal injury was associated with higher odds of acute symptomatic seizures [15]. Intraventricular hemorrhage is thought to cause seizures when associated with parenchymal injury, such as from periventricular hemorrhagic infarction, and is seen more commonly in preterm neonates [14, 16].

Prior studies have found that HIE to be the most common cause of acute provoked seizures [3], and although the incidence has decreased with therapeutic hypothermia, approximately half of children with HIE have seizures in spite of cooling [16]. In our entire cohort of infants with acute provoked seizures, 30% underwent therapeutic hypothermia, and amongst those with a radiologic diagnosis of HIE, 39% underwent hypothermia. This seemingly low rate of hypothermia can be explained by the fact that infants were enrolled into our study on the basis of EEG-confirmed seizures during the neonatal admission, not based on Sarnat [17] criteria for HIE and therapeutic hypothermia, and many of these seizures likely occurred beyond the 6 h window for therapeutic hypothermia initiation. Many of these infants with HIE as the underlying seizure etiology also had complex medical conditions that precluded them from meeting criteria for therapeutic hypothermia.

Unlike prior studies, in our study, focal ischemic stroke was also the most common radiologic etiology for acute provoked seizures. The total number of infants with HIE by radiologic diagnosis was lower than that by clinical diagnosis (28% vs 42%) and the total number of ischemic strokes by radiologic diagnosis was higher compared to clinical diagnosis (36% vs 26%). This is likely due to radiologic interpretation of focal areas of reduced diffusion as perinatal stroke, which in the clinical context of peripartum asphyxia, would clinically be assigned as sequela of HIE. One prior study suggested that up to 5% of children with neonatal encephalopathy presumed to be secondary to HIE actually had ischemic stroke on MRI, and thus the imaging diagnosis may be a more accurate reflection of the true etiology [18]. Prior research from our group on acute provoked seizures in neonates did not find a difference in seizure burden based on clinical etiology for seizures after adjusting for potential confounders [3], thus the differences in seizure burden based on radiologic etiology adds to existing knowledge and may inform counseling during the immediate hospitalization period.

Neonates with MRI evidence of intracranial infection, such as leptomeningeal enhancement or purulent reduced diffusion, had higher odds of abnormal neurologic examination at discharge compared to those without MRI evidence of infection. Neonatal bacterial or viral meningitis is known to cause significant morbidity, often leading to subsequent complications including stroke, vasculopathy, venous thrombosis, hydrocephalus, and/or cerebral abscess formation. Acute seizures in the setting of infection have been known to persist longer than other etiologies, possibly related to ongoing inflammation during treatment [14]. The poor short-term outcomes in this population may reflect the additional complications that arise secondarily as a result of infection.

Injury to the cortex, regardless of underlying etiology, was significantly associated with higher seizure burden and treatment refractory seizures. The cortex, which contains principal and interneurons that form networks of synaptic circuits throughout the brain, is subject to excitability, which is dysregulated in the setting of seizures [19]. Neonates with cortical injury also demonstrated lower odds of EEG-only seizures.

Deep gray injury was associated with an abnormal neurologic examination at discharge. These findings are consistent with existing neonatal HIE literature, as injury to these structures is strongly associated with adverse motor, cognitive and language outcomes in this population [20, 21]. Prior research from our group has shown that deep gray injury in neonates with acute symptomatic seizures was also significantly associated with subsequent development of infantile spasms later in life [22]. Thus, the identification of deep gray injury in this population has implications for not only for counseling on short term outcomes, but ramifications for long term care planning and early intervention.

Although we report imaging findings from a large cohort of neonates with acute provoked seizures enrolled prospectively from seven quaternary pediatric centers, our study has limitations. First, although the clinical MRIs were centrally reviewed by a pediatric neuroradiologist with expertise in neonatal imaging, they were acquired using heterogeneous imaging protocols that varied across institutions. As these protocols varied in available sequences and sequence parameters, it is possible that some abnormalities were variably detected based on available imaging. Second, we report only short-term outcomes; detailed evaluation of the association between MRI findings and childhood neurodevelopment in infants with acute provoked seizures is planned. Third, as neonates included in this study were initially enrolled as part of a larger study on long-term neurodevelopmental outcomes after acute symptomatic seizures, neonates who did not survive the neonatal admission were excluded, and the results of our study are not applicable in the setting of death prior to discharge from the neonatal hospitalization. Fourth, we did not investigate acuity of imaging injury, which is important to establish the timing of injury and highlights the importance of the maternal-placental-fetal triad and trimester-specific gene-environment factors in the development of neonatal central nervous system disease, including neonatal seizures [23]. Investigating additional gene-environment interactions and the maternal-placental-fetal triad will be an important aspect of future work in this field.

## Conclusion

In the context of neonatal acute provoked seizures, MRI findings of ICH or HIE as the underlying etiology for acute provoked seizures, and/or cortical location of injury were associated with high seizure burden. Radiologic evidence of infection and/or injury to the deep gray nuclei were associated with an abnormal neurologic examination at discharge. These findings on initial MRI can aid in parental counseling on short term outcomes for neonates with acute provoked seizures. Future work should characterize the association between brain MRI findings and neurodevelopmental outcomes after acute provoked neonatal seizures.

### Supplementary information

Supplementary Tables

## Data Availability

Multi-institutional data was collected as part of the Neonatal Seizure Registry and may be available upon request by contacting the corresponding author.
