# Supplementary material for: Neonatal brain MRI and short-term outcomes after acute provoked seizures
Source: J Perinatol. 2023 Jul 15;43(11):1392–7. doi: 10.1038/s41372-023-01723-3 (PMC10615741; doi:10.1038/s41372-023-01723-3)
Supplement: Supplementary file 1 — Supplementary Tables [file 41372_2023_1723_MOESM1_ESM.docx]

**Supplemental Tables**

**Supplemental Table 1.** Radiologic and clinical diagnoses for seizure etiology in 236 children with acute provoked seizures.

| **Diagnosis** | **Radiologic Diagnoses** | **Primary Clinical Diagnosis** |
| --- | --- | --- |
| Normal MRI | 22/236 (9%) | - |
| Hypoxic Ischemic Encephalopathy | 67/236 (28%) | 98/236 (42%) |
| Ischemic Stroke | 84/236 (36%) | 62/236 (26%) |
| *Arterial* | 53/84 (65%) | 47/62 (76%) |
| *Venous* | 10/84 (12%) | 8/62 (13%) |
| *Other/atypical* | 19/84 (23%) | 7/62 (11%) |
| Hemorrhage | 62/236 (26%) | 43/236 (18%) |
| Suspected Infection | 16/236 (7%) | 20/236 (8%) |
| Suspected Hypoglycemia | 8/236 (3%) | 4/236 (2%) |
| Other | 22/236 (9%) | 9/236 (4%) |
| Multiple diagnoses | 42/236 (18%) | - |

**Supplemental Table 2.** Odds of EEG-only seizures and treatment resistant seizures based on radiologic diagnosis and injury location in 236 children with acute provoked neonatal seizures.

| MRI Findings | EEG-only Seizures | | Treatment Resistant Seizures | |
| --- | --- | --- | --- | --- |
|  | Univariate* | Multivariate* | Univariate* | Multivariate* |
| Normal MRI  (N=22) | OR 2.2  [0.82-6.3], p<0.114 |  | ***OR 0.13[0.05-0.38], p<0.0001*** |  |
| HIE  (N=67) | OR 1.4  [0.2-2.8], p<0.42 |  | ***OR 5.2 [2.4-11.5], p<0.0001*** | ***OR 4.9 [2.2-10.8], p<0.0001*** |
| Ischemic Stroke  (N=84) | ***OR 0.42***  ***[0.52-0.9], p<0.03*** |  | OR 0.77 [0.4-1.4], p<0.38 |  |
| Intracranial Hemorrhage  (N=62) | OR 1.4 [0.7-3.0], p<0.35 |  | OR 1.2 [0.6-2.3], p<0.58 |  |
| Infection  (N=6) | OR 1.0 [0.28-3.9], p<0.96 |  | OR 2.3 [0.64-8.5], p<0.20 |  |
| Other diagnosis  (N=51) | OR 1.3 [0.6-2.9], p<0.46 |  | ***OR 0.30 [0.15-0.59], p<0.0001*** |  |
| Cortical Injury (N=118) | ***OR 0.2 [0.09-0.45], p<0.0001*** | ***OR 0.2 [0.09-0.45], p<0.0001*** | ***OR 2.6 [1.5-4.7], p<0.001*** | ***OR 4.9 [2.2-10.8], p<0.001*** |
| Deep Gray Injury (N=89) | OR 0.64 [0.3-1.3], p<0.22 |  | OR 1.8 [0.98-3.1], p<0.06 |  |
| Other Injury Location (N=61) | ***OR 3.2 [1.5-6.4], p<0.002*** |  | OR 0.86 [0.46-1.6], p<0.63 |  |

*Adjusted clinical confounders of sex, preterm birth and complex medical course
